# Phoneme Monitoring in Developmental Dyslexia: Pupillometric Evidence for Cognitive Rather than Acoustic Origins of Phonological Deficits

**DOI:** 10.3390/brainsci16070697

**Published:** 2026-06-30

**Authors:** Marina Rossi, Massimiliano Canzi, Tamara V. Rathcke

**Affiliations:** Department of Linguistics, University of Konstanz, 78457 Konstanz, Germany

**Keywords:** developmental dyslexia, phoneme monitoring, pupillometry, cognitive resources, acoustic salience

## Abstract

**Background.** Developmental dyslexia (DD) is a neurodevelopmental disorder characterized by persistent difficulties acquiring fluent reading. A core feature is impaired phonological processing, though its etiology remains debated. Two competing accounts attribute phonological deficits either to reduced acoustic sensitivity to lexical stress cues or to insufficient cognitive support during phoneme processing. **Methods.** To test these accounts, 57 Italian children (28 with DD, 29 typically developing) completed a phoneme monitoring task in which targets appeared in strong or weak syllables, varying in acoustic salience. A composite acoustic salience factor was derived from target cue properties, and an individual cognitive factor was computed from IQ, working memory, and shifting attention. Pupillometry was used to assess auditory sensitivity to acoustic salience and cognitive effort in real time. **Results.** The results showed that, behaviourally, children with DD showed significantly lower target identification accuracy and d’-sensitivity. However, pupil dilation during target processing did not differ between the two groups, while children with DD showed reduced pupillary responses on trials involving distractor rejection and missed targets. These physiological patterns correlated primarily with individual cognitive scores rather than acoustic salience. **Conclusions.** Taken together, these findings point toward an important role of individually varying cognitive resources during phonological processing and highlight the value of pupillometry as a sensitive, real-time index of cognitive engagement during a phonological task.

## 1. Introduction

Reading is a fundamental skill essential for success in education, employment, media engagement, and accessing critical services [1,2]. Notably, estimates suggest that between 3% and 10% of the general population is affected by developmental dyslexia (DD) [3,4]—a neurodevelopmental condition that leads to persistent difficulties in acquiring literacy skills [5]. While the precise etiology of this condition remains a topic of ongoing debate, it has become increasingly recognized that DD encompasses large variability—and partly insufficient ability—in linguistic, cognitive, sensory and motor skills [6,7]. One of the most consistently reported deficits in DD is phonological in nature [8,9,10,11]. The development of well-formed phonological representations, along with the ability to actively use and manipulate them, is a process known as phonological awareness [12,13]. Phonological awareness is essential for reading acquisition [14,15,16]. Previous studies have shown that the ability to discriminate, identify, manipulate, or classify speech sounds into specific categories may be weaker in individuals with DD [8,10,17,18,19,20,21,22]. However, some of the previously deployed phonological awareness tasks may tap cognitive abilities, such as working memory, attention, and executive functions [23,24,25,26]. For example, succeeding at the spoonerism task that requires swapping initial sounds of two words (e.g., turning *ball* and *tin* into *tall* and *bin*) draws on several cognitive operations: focusing on the two words, identifying and segmenting their initial phonemes, holding these phonemes and the rhymes in working memory, and then exchanging them.

Existing evidence suggests that the cognitive abilities implied in phonological awareness tasks are frequently impaired in individuals with DD [27,28,29,30]. For example, there is some indication that phonological awareness deficits scale with working memory limitations in both typically developing children aged 9–10 years and those with dyslexia [28]. Moreover, adults with DD display difficulties with the perception of voiceless vs. voiced stops, especially under conditions of high stimulus complexity, possibly due to increased working memory demands [30]. Our recent study [31] provided evidence for a potential dissociation between phonological processing (measured as the ability to identify a specified phoneme embedded in a nonce-word) and the use of attentional resources (measured as the attentional shifting ability) in adolescents aged 14–19 years with DD as compared to their typically developing peers: while higher levels of shifting attention were associated with better phoneme identification performance in typical development, this relationship was absent in the DD group, showing consistently low identification accuracy regardless of attentional shifting abilities [31]. At the same time, cognitive profiles in DD are highly heterogeneous, both in the presence and type of a deficit. Studies of dyslexic children (aged 8–13 years) consistently reveal subgroups: some children show no measurable cognitive impairment [32], while others divide into distinct cognitive profiles, with deficits in either working memory, divided attention, attentional shifting, or vigilance [33,34,35,36]. Critically, findings from studies with adults suggest that group-level cognitive differences may diminish once IQ is controlled for [36], pointing to an age-related compensation as a potential source of cross-study inconsistency. Overall, cognitive heterogeneity in DD points toward an interaction between deficit profile, developmental stage, and individual IQ.

Alternatively, issues with phonological representations may also stem from other impairments, e.g., a reduced auditory sensitivity to acoustic cues, especially those encoding lexical stress [10,37]. For instance, Leong, et al. [38] and Goswami, et al. [39] found that English-speaking children with DD aged 9–13 years performed worse than their typically developing peers on tasks examining the perception of the location of lexical stress and showed auditory deficits linked to impaired syllable rise-time processing. Reduced sensitivity to lexical stress has also been observed in languages with more transparent orthographies than English, e.g., Spanish and Italian [40,41,42]. These findings have jointly contributed to the Temporal Sampling Framework [37], which proposed that DD arises from a misalignment between syllable rise-times and neural oscillations in the delta range, i.e., 0.5–3 Hz [43,44]. This misalignment could then disrupt the encoding of stressed (i.e., strong) vs. unstressed (i.e., weak) syllables and thus impede the extraction of acoustic-phonetic information essential for phonological processing.

However, research on lexical stress processing in individuals with DD has yielded mixed results, frequently interpreted through the lens of the cognitive load and metalinguistic awareness hypothesis [45,46,47]. Along these lines, a recent meta-analysis by Mundy and Wood [47] examined 124 effect sizes drawn from 37 studies, involving 1771 participants (mean age = 13.7 years). The analysis revealed a moderate-to-large deficit in prosodic processing among individuals with DD compared to chronological-age-matched controls (Hedges’ *g* = −0.70). However, this impairment was only pronounced in tasks requiring explicit processing of prosodic features and tapping the ability to consciously analyze and manipulate prosodic information. These included tasks in which participants were asked to locate stressed syllables, make same/different judgments about stress patterns, and correct mispronounced stress. In contrast, no significant group differences were observed for tasks involving implicit prosodic representations, which assess the implicit encoding of prosodic features without requiring any explicit, metacognitive engagement. Moreover, larger effect sizes were generally reported in studies using less transparent orthographies (predominantly English), although this finding may reflect a sampling bias, given the predominance of English-language studies. Overall, evidence in favor of generally reduced sensitivity to lexical stress remains limited, particularly in languages with transparent orthographies. For example, English readers may need to rely more heavily on prosodic and/or other global cues to correctly process words with irregular and less transparent grapheme-to-phoneme mappings (e.g., <ough> in *through*, *though*, *tough*, *cough*, *hiccough*), whereas Italian readers can generally use stable grapheme-to-phoneme correspondences with less reliance on prosodic analysis to decode words [15].

This gap in the literature calls for methodologies capable of capturing subtle, time-sensitive indicators of phonological and acoustic processing. To this end, pupillometry provides an established method of measuring variations in pupil size over time as a real-time physiological index of cognitive processing, auditory sensitivity, and cognitive effort [48,49,50]. Changes in pupil size over time respond dynamically to a range of external and internal cognitive events, including phonological processing and acoustic sensitivity [50,51,52,53,54,55,56,57,58,59,60]. Previous research has demonstrated that pupil size increases when listeners experience acoustically more salient auditory stimuli, suggesting a direct link between pupil dilation and auditory processing of acoustic salience [51,55,60]. An increase in acoustic salience has been previously encoded by a local increase in intensity [55], a substantial change in fundamental frequency [60], and phonologically illicit or otherwise deviant phonetic realizations [51]. At the same time, pupil dilation has been consistently associated with cognitive processing and load [49,50,54,56,57,58,59]. Several studies have highlighted the relationship between pupil dilation and attention [49,54], working memory capacity [58], arousal and cognitive engagement [56,57,59]. Specifically, greater dilation has been observed in tasks requiring high working memory load, e.g., maintaining and manipulating multiple vs. isolated items [58] and also tasks examining attentional abilities [54] and degraded speech processing [59]. When task demands exceed available resources or when task engagement declines, pupil dilation may plateau or decrease, indicating cognitive overload or disengagement [56,58]. Pupil size variations thus appear to reflect the allocation of cognitive resources to the task at hand. These cognitively driven changes are typically smaller than those induced by light-to-dark transitions via the pupillary light reflex, generally falling within the range of 0.01–0.5 mm, in contrast to 2–8 mm for luminance-related responses [50].

As pupillary responses unfold over time [48,61], this method enables tracking of the temporal dynamics of cognitive processing during a phonological task similar to electrophysiological measures, while being less time-consuming and more comfortable to the participant as compared to cortical measures. As discussed in previous work, pupillometry and EEG share the ability to capture covert cognitive activity, though their timing relative to behavioral responses varies [48,61].

The activation latency of a pupillary response to an auditory stimulus is typically found around 500 ms after the stimulus onset, with peak dilation reached between 1 and 1.4 s [49,52,53]. In contrast, EEG responses such as the N1 or P2 components emerge much earlier (typically within 100–250 ms), reflecting rapid neural encoding [62]. Regardless of these differences in temporal dynamics, pupillometry has been established as a reliable and non-invasive means of investigating phonological processing, along with its underlying cognitive and acoustic mechanisms [50,51,52,53,54,55,57,58,59,60]. By capturing responses across the full task duration, pupillometry can complement binary behavioral measures and help to measure the perception of acoustic salience and the levels of cognitive effort during auditory processing [4,63]. Despite these methodological advantages, very few studies have deployed pupillometry to study phonological abilities and their underlying issues in listeners diagnosed with DD.

Given the ongoing debate about the underlying causes of DD [29,30,31,37,38,41,64], the present study used pupillometry to investigate the role of auditory sensitivity to speech acoustics vs. cognitive resources during phonological processing in children with and without DD. The central research question of the study was whether phonological difficulties in DD result primarily from reduced sensitivity to acoustic–prosodic salience or from inefficient domain-general cognitive support during phonological processing [65]. To extend existing evidence to languages with shallow orthographic depth [47,66], the study focused on Italian [67,68]. Italian is a language with phonologically relevant lexical stress contrasts, which can be acoustically marked by duration and possibly fundamental frequency when words are spoken in isolation [41,65,69,70]. It is an example of a language with more shallow and transparent grapheme-to-phoneme mappings than English [71]: that is, a letter in Italian is always pronounced consistently across all words it occurs in (e.g., <a> in *casa*, *mare*, *padre* is always pronounced as /a/), meaning that, unlike English, Italian orthography can generally be decoded through stable grapheme-to-phoneme correspondences [72]. The study tested two hypotheses:

1. The *Auditory Sensitivity Hypothesis* [37,38,41,64] proposed that dyslexic deficits in phonological processing stemmed from reduced auditory sensitivity to prosodic cues encoding lexical stress. Accordingly, we expected that Italian children with DD would demonstrate lower sensitivity (along with lower pupillary responses) to the variability in acoustic salience arising from the combination of acoustic cues known to encode lexical stress in Italian and other languages, i.e., duration, intensity, and fundamental frequency [65,73].
2. The *Cognitive Mediation Hypothesis* [29,30,31] proposed that phonological processing was mediated primarily by individual cognitive abilities. Accordingly, we expected that the group of Italian children with DD would have lower levels of general cognitive abilities (as measured by IQ) and cognitive resources (including working memory and shifting attention) and show smaller pupil dilation during phoneme identification, reflecting diminished cognitive support for phonological processing in DD [31].

## 2. Method

### 2.1. Participants

Fifty-seven Italian monolinguals from different schools in the Milan metropolitan area and its hinterlands volunteered as participants in this study. The participating schools typically attracted pupils from households with middle to high socio-economic backgrounds. Twenty-eight children (19 males), aged 8 to 10 years, had a formal diagnosis of DD, as described in the individual reports provided by the schools. The diagnosis was performed by a trained team of psychologists and based on the criteria outlined in the *Diagnostic and Statistical Manual of Mental Disorders*, *Fifth Edition* [5]. Associated developmental issues such as dyspraxia, ADHD, speech and language delays or impairments were treated as exclusion criteria. Since dyslexic participants with and without dyscalculia exhibit comparable phonological profiles [11,31,74], dyscalculia was not treated as an exclusion criterion and occurred in four participants with DD. A separate group of 29 children (19 males) with typical development (TD) in speech, language, and literacy acquisition served as the control group. Both TD and DD exhibited a comparable age (TD group, *Mdn* = 10, *M* = 9.3 (0.77), range = 8–10; DD group, *Mdn* = 10, *M* = 9.4 (0.74), range = 8–10; *W* = 418, *p* = 0.83) as measured by the Wilcoxon Rank-Sum test [75,76]. To participate in this study, participants’ IQ had to fall within the normal range, assessed using Raven’s Colored Progressive Matrices—a 36-item nonverbal reasoning task with multiple-choice responses [77,78]. Scores were normalized into percentile ranks [77].

Among the recruited volunteers, two children had eye pathologies, meaning that pupillometry could not be adequately performed [79] and leading to their exclusion from the dataset. The analyses reported below are therefore based on the data collected from 55 participants in total, including 28 DD participants (19 males) and 27 TD participants (19 males).

### 2.2. Stimuli

The stimuli of the study consisted of 64 polysyllabic nonce-words, 50% of which contained a target phoneme, represented either by an obstruent [t] or a sonorant [l], either as a singleton [t], [l] or a geminate [tː], [lː] (e.g., [sekapodelavi] vs. [sepumarulːasi], [makaralutani] versus [mefusotːaduri]). The target phonemes were distributed across strong syllables (25%) and weak syllables (75%). This design enabled a more detailed investigation of targets in weak syllables, which tend to show larger group differences between individuals with and without DD than strong syllables, at least in English [80]. Targets did not occur in the very first or last syllable. The nonce-words consisted of 6 to 8 open syllables (CV) [81]. The duration of stimuli ranged between 843 and 1322 ms, with target onsets occurring in the time window between 116 and 900 ms from the start of each nonce-word. Nonce-words without the targets contained other sonorants and obstruents, as either singletons or geminates.

### 2.3. Experimental Task

An Italian version of the phoneme monitoring task [80,82,83,84,85,86] was developed to make it suitable for children. Specifically, stimulus length, task duration, and monitoring demands were reduced compared to adult versions of the task in order to facilitate administration in school-aged children [31]. The task required participants to listen to spoken nonce-word stimuli and respond whenever they detected a specified target phoneme. The 64 stimuli were presented in four blocks, each containing eight targets and eight non-targets presented in random order with an interstimulus interval of 1800 ms. At the start of each trial, instructions related to the target that had to be monitored in the upcoming block were provided in both written and oral formats. Participants were instructed to focus on one target type at a time. High-quality headphones (Sennheiser HD 380, Sennheiser, Wedemark, Germany) were used for stimulus playback, and the volume was set at an individually comfortable level.

Prior to the start of the phoneme monitoring task, each participant was asked to wear Tobii Pro Glasses 3 and follow the system’s default built-in one-point calibration, which accounts for changes in pupil diameter during the task [87]. The Tobii Pro Glasses 3 were connected to the relevant software running on a Lenovo Thinkpad X1 Carbon (Lenovo, Morrisville, NC, USA) via an ethernet cable. An internal algorithm calculated eye position and pupil size by capturing images of participants’ eyes at a sampling frequency of 50 Hz [87]. Efforts were made to maintain a consistent viewing distance of approximately 0.7 m from the monitor and to control artificial overhead lighting, avoiding direct light sources, which would cause reflections on the computer display. Behavioral responses to the phoneme monitoring task were collected via PsychoPy (version 3.3)—a software that recorded participants’ responses in conjunction with their reaction times [88]. To avoid technical conflicts with the pupillometry software, the PsychoPy experiment ran on a separate laptop, a MacBook Air (Retina, 13-inch, Apple Inc., Cupertino, CA, USA, 2018). The recordings of the two experimental laptops were synchronized by using a tone, which was played at the start of the experiment through PsychoPy and recorded via the eye tracker’s microphone.

Participants had to listen to all nonce-words while looking at a cross symbol in the center of the laptop screen. They were instructed to press the space bar when they detected the target phenome while keeping their hand on the space bar key throughout the experiment. A familiarization phase, in which participants practiced monitoring for the target phoneme /f/ (not included in the main experiment), provided an opportunity for participants to pilot their understanding of the task, ask questions and indicate when they were ready to proceed. All participants were individually tested at their respective schools, with each session lasting approximately 90 min. Breaks were provided as needed. Consent forms were collected prior to testing. An experimental session began with the phoneme monitoring task and concluded with cognitive and other tasks not part of the present study. Ethical approval for the study was granted by the Ethics Board of the University of Konstanz (IRB statement 05/2021).

### 2.4. Cognitive Assessment

In order to examine and compare individual variability in cognitive resources [30,89,90], the present study measured working memory, shifting attention, and non-verbal IQ in all participants. Working memory was defined as the capacity to temporarily store and manipulate information during ongoing cognitive processing [91]. It was assessed using the *Letter-Number Sequencing* subtest from the WISC-IV [92,93], which involved listening to, ordering, and repeating mixed series of letters and numbers that were presented orally. Shifting attention was the ability to flexibly redirect attentional focus between stimuli, tasks, or mental operations [94]. It was measured using the NEPSY-II *Response Set* subtest [95,96] in which participants had to listen to a pre-recorded list of words and touch a colored circle when either a matching or a non-matching color was heard, e.g., *“touch the yellow circle when you hear the word red”* [97,98]. This Stroop-like task assesses the ability to retain and shift attentional focus on complex response sets while inhibiting automatic responses [95]. Scores obtained in these tests were transformed into standard scores (*M* = 10, *SD* = 3) based on Italian normative data from the previous literature [92,96]. Finally, non-verbal IQ was defined as general cognitive ability of visual pattern recognition, spatial reasoning, and abstract problem-solving, independent of linguistic skills [99]. It was assessed using *Raven’s Colored Progressive Matrices*, which required participants to select the missing piece that best completed a visual pattern from multiple options [77]. For all tests, child-specific versions were used.

**Figure 1:**
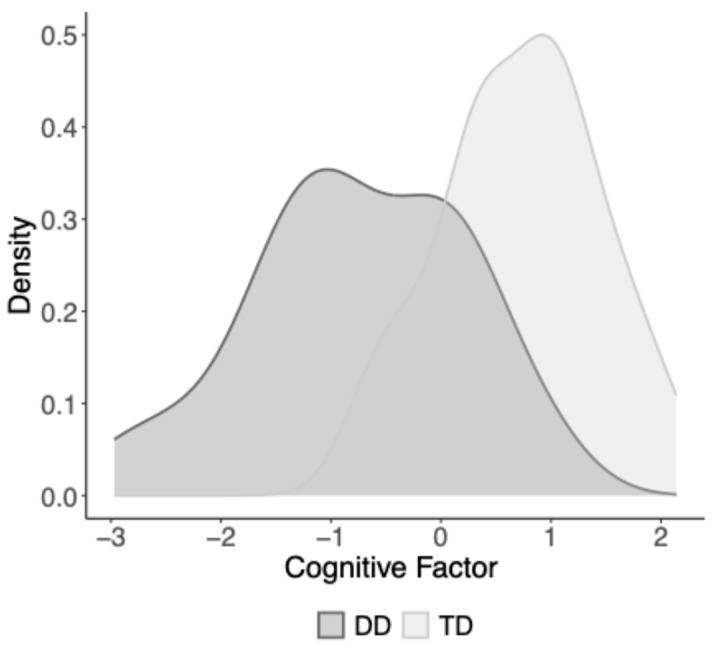
Density distribution of the cognitive factor across groups. The dark gray distribution represents the dyslexic group (DD), and the light gray distribution represents the typically developing group (TD). The factor was derived from IQ, working memory, and shifting attention scores; higher values indicate stronger performance.

Given that cognitive abilities are often interrelated [100,101] and phonological processing relies on multiple shared cognitive resources rather than isolated functions [26,27,28], a factor analysis was conducted using the R package *psych* [102], employing promax rotation and Bartlett’s method for factor score estimation. The analysis identified latent factors that explained the patterns of covariance among observed cognitive scores, with the underlying idea that if cognitive scores correlated strongly with each other, they may share a common underlying construct [103]. The statistic supported a single-factor analysis, which accounted for 51% of the total variance (SS loadings = 1.54). Factor loadings were as follows: *IQ* = 0.57, *shifting attention* = 0.74, and *working memory* = 0.81, with corresponding uniqueness values of 0.67, 0.45, and 0.34. Accordingly, *shifting attention* and *working memory* were strongly correlated with each other (*r* = 0.61, *p* < 0.001), and both were moderately correlated with *IQ* (*r* = 0.43, *p* = 0.001 and *r* = 0.47, *p* < 0.001, respectively). Therefore, one factor score was derived from the factor analysis and used as an index of each participant’s general cognitive profile in the statistical models reported below. A linear regression model confirmed that the cognitive factor was significantly lower in the DD group compared to the TD group, even though—as shown in Figure 1—there was a substantial overlap in the DD vs. TD values of the cognitive factor [32,34,104].

### 2.5. Data Pre-Processing and Statistical Analyses

**Stimulus acoustics.** To examine the role of acoustic factors on the correct identification of phoneme targets, we extracted the duration of each target and its preceding vowel (in ms), target intensity (in dB), and fundamental frequency (F_0_, in Hz) of the vowel following the consonant target in PRAAT [105]. The extracted acoustic measurements underwent a normalization process, using the sentence average as a baseline [106]. The target duration and intensity were normalized by dividing each respective value by the average syllable duration or the average syllable intensity. Duration ratios were additionally log-transformed. Higher positive values indicated an increase relative to the baseline duration and intensity. The F_0_ of the following vowels was normalized using the semitone conversion [107], with the baseline defined as the average F_0_ of the sentence [108]. The procedure resulted in values around 0, indicating no F_0_ deviation, negative values indicating a fall, and positive values indicating a rise relative to the baseline. Given that the acoustic measures were likely to covary [109,110] and show cue redundancy [111], we performed a factor analysis. The analysis based on Bartlett’s method and the promax rotation from the R-package *psych* [105] established that one factor was sufficient to capture the variability in the acoustic data, accounting for 41% of the total variance (SS loadings = 1.23). The factor had the following loadings: F_0_ (0.95), intensity (−0.38), and duration (−0.43), with their uniqueness being 0.85, 0.10, and 0.82 respectively. While normalized intensity was negatively correlated with both normalized F_0_ (*r* = −0.37, *p* = 0.04) and duration (*r* = −0.41, *p* = 0.02), the correlation between F_0_ and duration was negligible and not statistically significant (*r* = 0.16, *p* = 0.37). The derived factor was implemented in the data analysis as a measure of the acoustic salience of a target embedded in an experimental stimulus [110].

**Responses.** To derive the individual d’-sensitivity index [112], the total number of correct responses (n-hit), missed responses (n-miss), false alarms (n-fa), and correct rejections (n-cr) were extracted from the raw PsychoPy data. Subsequently, the total number of each response per participant was input into the d’-prime function from the R-package *psycho*, which derived d’-sensitivity as an index of a person’s ability to distinguish between signal and noise in a detection task [112]. A higher d’-value indicates better discrimination between targets and distractors, while a lower d’-value reflects reduced sensitivity. In addition, we measured response accuracy by coding all correct responses as 1 and missed or anticipated responses as 0.

**Pupillometry.** Raw pupillometry data were extracted using Tobii Pro Lab software (v.1.207, [87]), with average pupil size recorded at a sampling rate of 50 Hz (i.e., every 20 ms). As timestamps were initially indexed in units (e.g., 1, 2, 3…), they were subsequently converted to milliseconds by multiplying by 20. We time-synchronized these data with the behavioral responses collected through PsychoPy by using the time stamp of a tone played through PsychoPy and aligning it to the time stamp of the same tone recorded by the eye tracker’s microphone. Missing data (e.g., due to blinks) were automatically excluded by Tobii Pro Lab software during the experiment, making blink labeling [48] and interpolation [61] unnecessary. In the analyses reported below, only trials with no more than 30% missing data were considered [113,114].

Following Mathôt [50], pupil data were baseline-corrected to reduce inter-individual variability in tonic pupil size. Baseline-corrected pupil diameter (BCPD) was obtained by correcting pupil-size recordings relative to a pre-stimulus baseline. For trials involving targets, the average pupil size during the 500 ms preceding target onsets was subtracted from subsequent values, enabling comparison of task-evoked dilation between correctly identified and missed targets. For trials without a behavioral response, the same correction procedure was applied, but using the 500 ms preceding word onset (rather than target onset) as the baseline. This distinction ensured appropriate temporal alignment with stimulus type across conditions and allowed for reliable comparison of pupil dynamics between correctly rejected non-targets and missed targets.

**Statistical analyses.** All analyses were conducted in RStudio 2025.01 [115] running R version 4.3.1 [116] and using the following R packages: *tidyverse* [117], *psycho* [118], *psych* [102], *lmerTest* [119], and *lme4* [120]. Generalized additive mixed models (GAMMs) were fitted using the *mgcv* package [121], with model comparison functions taken from the *itsadug* package [122].

To analyze behavioral responses, linear mixed-effects regressions (LMMs) were fitted to d’-values, while logistic mixed-effects regressions were fitted to the accuracy data containing binary [120] correct (1) vs. incorrect (0) responses. Full models tested for an interaction between *group* (TD vs. DD) and *cognitive factor*. Models of accuracy additionally included an interaction between *group* and *acoustic salience*. To account for variability across *stimuli* and *participants*, both were included as random effects in all models. Maximal random-effects structure was tested and retained if the models converged and did not produce singular fits [123]. A BOBYQA optimizer helped to combat singular-fit issues of logistic mixed-effects regressions [124]. Maximum likelihood estimation was applied. The best-fitting models were obtained using a backward-fitting procedure. Initially, all interactions of interest were included, and non-significant interactions were subsequently removed stepwise based on the likelihood-ratio test [120].

To examine the relationship between pupil dilation and behavioral markers—in our case, phoneme identification ability, cognitive resources, and acoustic salience [49,51,54,55]—we first examined correlations between d’-sensitivity, cognitive factor, and acoustic salience factor, on the one hand, and task-evoked pupil responses, on the other hand. For this, pupil data recorded during processing of stimuli containing correctly identified targets and correctly rejected non-targets were averaged within a time window defined from the onset of pupil dilation until its return to the baseline (this ensured that the measurement of pupil dilation was comparable across the two types of stimuli). The average dilation value was then correlated with each behavioral marker, using the full response window. It provided a stable global measure for correlational analyses independently of peak-latency variability [50,59].

To test the hypotheses, we first fitted GAMMs to pupil dilation responses in order to examine the time course of pupillary responses to target embeddings, testing for an interaction between *group* (TD vs. DD) and *condition. Condition* contrasted responses to (1) correctly identified vs. missed targets, and (2) correctly rejected control stimuli vs. missed targets. Subsequently, we ran complementary LMMs on subject- or stimulus-averaged BCPD values. All interactions were defined both parametrically and through smooth terms. A reference smooth for *time* was included, along with difference smooths for each level of *condition*. Random intercepts for *participants* and *stimuli* were included to account for variability associated with individuals and items. Models were fitted using maximum likelihood. Smoothing parameters (k-values) were assessed using the gam.check() function to ensure an adequate model complexity. Best-fitting GAMMs were identified through model comparisons by evaluating the fit of the full model against successively simplified models as well as the null model (i.e., a model with only an intercept and no predictors), using the compareML() function [125]. The significance and shape of smooth terms were evaluated by (a) inspecting the estimated smooths with plot_smooth(), and (b) analyzing factor differences for *group* or *condition* in smooth terms using plot_diff() [125,126,127]. Restricted maximum likelihood estimation was applied to the best-fitting model to obtain unbiased parameter estimates [128].

Additional LMMs tested average BCPD values computed for each participant–stimulus trial as a function of condition. These models included random intercepts for *participants* and *stimuli* to account for repeated measurements within individuals and the reuse of the same stimuli across participants. Fixed-effect predictors included two three-way interactions of (1) *group* (TD vs. DD), *condition* (correctly identified vs. missed targets, or correctly rejected control stimuli vs. missed targets), and *acoustic salience* (numerical predictor scaled around the mean), and (2) *group*, *condition*, and *cognitive factor* (numerical predictor scaled around the mean). Mean BCPD values were extracted within the inter-peak interval, corresponding to the task-evoked dilation window defined by the response peaks following peak-centered approaches; this restriction isolates the stimulus-evoked component and preserves temporally localized effects [50,129,130]. These additional analyses allowed us to test whether mean pupil dilation could be predicted by stimulus-level acoustic salience, in line with the *Auditory Sensitivity Hypothesis*, or by a composite cognitive factor, in line with the *Cognitive Mediation Hypothesis*.

## 3. Results

### 3.1. Analyses of Behavioral Responses

**Figure 2:**
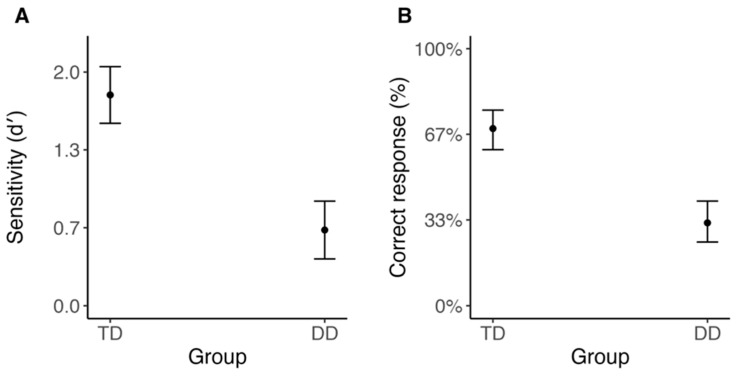
Group differences in phoneme monitoring performance. Estimated group-level differences between participants with developmental dyslexia (DD) and typically developing participants (TD) are shown for (**A**) d’-sensitivity and (**B**) accuracy.

**Sensitivity d’.** On average, the TD group correctly responded to approximately 71% of the stimuli containing the target embeddings and to 87% of the control stimuli. In contrast, participants with DD correctly responded to 42% of the target embeddings and 80% of the control stimuli. Linear mixed-effects model best-fitting individual d’-values revealed a significant main effect of *group* (*F*(1,55) = 42.82, *p* < 0.001, see Figure 2A), suggesting that participants with DD had a significantly lower d’-sensitivity (*predicted M* = 1.80) than TD participants (*predicted M* = 0.65, *β* = 1.16, *SE* = 0.18, *t*(55) = 6.54, *p* < 0.001). In contrast, *cognitive factor* was not significant either in interaction with *group* (*F*(1,53) = 0.09, *p* = 0.75) or as a main effect (*F*(1,54) = 1.28, *p* = 0.26).

**Accuracy.** Logistic mixed-effects model best-fitting accuracy data also retained one main effect of *group* (χ^2^(1) = 38.54, *p* < 0.001, see Figure 2B), indicating that phoneme monitoring accuracy was significantly lower in participants with DD (*predicted M* = 0.33) than in those with TD (*predicted M* = 0.68, *β* = 1.54, *SE* = 0.21, *z* = 7.31, *p* < 0.001). Neither *cognitive factor* (χ^2^(1) = 0.87, *p* = 0.34) nor *acoustic salience* (χ^2^(1) = 0.12, *p* = 0.72) interacted significantly with *group*. There was also no significant main effect of either *cognitive factor* (χ^2^(1) = 0.25, *p* = 0.13) or *acoustic salience* (χ^2^(1) = 2.07, *p* = 0.15).

### 3.2. Analyses of Pupil Responses

**Figure 3:**
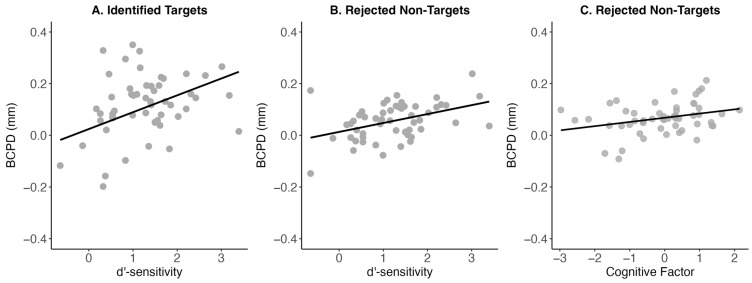
Correlations between behavioral measures and pupil dilation. Graphs display individual data points (gray dots) and fitted regression lines for (**A**) individual d′-sensitivity scores and baseline-corrected pupil diameter (BCPD, in mm) during correct target identification (250–1500 ms after target onset); (**B**) d′-sensitivity and pupil dilation during correctly rejected non-targets (250–2000 ms after nonce-word onset); (**C**) the cognitive factor (derived from IQ, working memory, and shifting attention scores) and pupil dilation during correctly rejected non-targets.

**Correlations for correctly identified target trials.** BCPD in response to strings containing correctly identified targets was significantly and positively correlated with participants’ d’-scores, *r*(53) = 0.39, *p* = 0.003, 95% CI [0.14, 0.59], suggesting a linear relationship between phonological processing and pupil dilation. As indicated in Figure 3A, greater pupil dilation was associated with higher d’-sensitivity scores. However, no correlations were found for the pupil dilation in response to correctly identified targets and either *cognitive factor* (*r*(53) = 0.06, *p* = 0.64, 95% CI [−0.20, 0.32]) or *acoustic salience* (*r*(901) = −0.023, *p* = 0.48, 95% CI [−0.08, 0.04]).

**Correlations for correctly rejected target trials.** The analysis based on BCPD during the processing of correctly rejected non-targets also revealed a strong positive correlation with d′-scores (*r*(53) = 0.40, *p* = 0.002, 95% CI [0.15, 0.60]), further corroborating the relationship between phonological processing and pupil dilation. As shown in Figure 3B, greater pupil dilation during correctly rejected non-targets was associated with higher d’-sensitivity scores. Additionally, there was a significant positive correlation with *cognitive factor* (*r*(53) = 0.29, *p* = 0.04, 95% CI [0.02, 0.51], see Figure 3C), indicating that lower levels of the cognitive factor were associated with smaller pupil dilation and vice versa.

**Hypothesis testing (GAMMs for correctly identified targets vs. missed targets).** The best-fitting GAMM of BCPD (time-locked to target onset for correctly identified targets vs. missed targets) retained the parametric interaction between *group* and *condition* along with their smooth terms (i.e., the reference smooth for *time* and the difference smooths of *time* for each level of the group-by-condition factor). GAMM diagnostics confirmed model convergence and adequate smoothness (all k-index values ≈ 0.99; all *p*-values > 0.05). Model comparisons using maximum likelihood confirmed that this model provided a superior fit relative to the null model (ΔAIC = −327.13, χ^2^(9) = 162.54, *p* < 0.001), and compared to a simplified model including only *group* (ΔAIC = −172.98, χ^2^(9) = 196.77, *p* < 0.001) or including only *condition* (ΔAIC = −90.59, χ^2^(9) = 114.38, *p* < 0.001). Both single-predictor models provided significantly better fits than the null model (only *group* model: ΔAIC = −3.99, χ^2^(3) = 3.99, *p* = 0.047; only *condition* model: ΔAIC = −141.36, χ^2^(3) = 141.36, *p* < 0.001), indicating that both *group* and *condition* accounted for some variance in pupil responses. Nevertheless, neither single-predictor model was sufficient to fully capture the data, as both were clearly outperformed by the model including the interaction. A model including additive effects of *group* and *condition* also improved model fit, but was still outperformed by the model including the interaction (χ^2^(3) = 19.37, ΔAIC = −43.65, *p* < 0.001).

**Figure 4:**
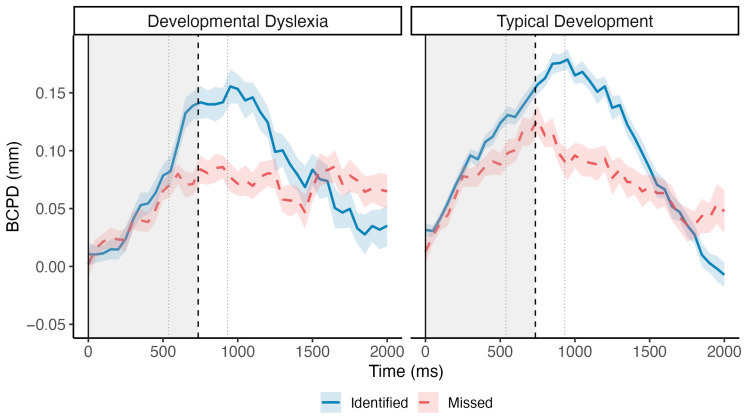
Time course of baseline-corrected pupil diameter (BCPD, in millimeters) for correctly identified vs. missed targets. The graph displays blue curves for correctly identified targets and red curves for missed targets. The left-hand panel shows results for participants with developmental dyslexia, the right-hand panel for typically developing participants. The x-axis is aligned to the target onset (0 ms). The solid vertical line marks the target onset, the dashed vertical line marks the average target offset, and the dotted vertical lines represent ±1 standard deviation of the target offset. The gray shaded area indicates the average target duration.

Subsequent comparisons of the fitted curves (using the plot_diff() function in R, [121,122]) revealed no differences between the TD and DD groups within either condition: pupil responses to identified trials did not differ between groups, nor did responses to missed trials. In contrast, identified trials elicited larger pupil dilations than missed trials in both groups. However, the temporal dimension of these responses differed between the two groups. In the TD group, the divergence between identified and missed responses emerged earlier (before ~500 ms) and remained pronounced beyond 1500 ms. In the DD group, the separation between conditions emerged later and was temporally more restricted. Peak estimates further supported this temporal difference, showing delayed responses in the DD group relative to the TD group (identified targets: TD = 936 ms, BCPD = 0.168 mm; DD = 959 ms, BCPD = 0.129 mm; missed targets: TD = 728 ms, BCPD = 0.118 mm; DD = 763 ms, BCPD = 0.088 mm). A visual inspection of the fitted trajectories (see Figure 4) further highlighted group differences in the dynamics of missed trials. In the TD group, missed responses followed a bell-shaped trajectory similar to that of identified responses, though reduced in magnitude. In the DD group, however, missed responses appeared flatter and more temporally diffuse. Taken together, these findings indicate that the significant interaction between *group* and *condition* is driven primarily by delayed peak latency in the DD group and by flatter, less temporally differentiated dynamics of missed responses in DD, rather than by differences in the overall magnitude of pupil responses.

**Hypothesis testing (LMMs for correctly identified targets vs. missed targets).** Additional linear mixed-effects analyses based on BCPD averaged by participant and stimulus and extracted for each peak interval (i.e., 300 ms time window around the observed peak latency, cf. [50,129,130]) provided converging evidence for the GAMM results. The best-fitting LMM retained a significant main effect of *condition* (χ^2^(1) = 11.87, *p* < 0.001), indicating larger pupil dilations for correctly identified than for missed targets. In contrast, neither the main effect of *group* (χ^2^(1) = 0.78, *p* = 0.38), nor the interaction of *group* and *condition* (χ^2^(1) = 0.11, *p* = 0.74) significantly predicted mean pupil dilation in these data. Furthermore, no significant interactions emerged for *group*, *condition*, and *cognitive factor* (χ^2^(1) = 0.90, *p* = 0.34) or for *group*, *condition*, and *acoustic salience* (χ^2^(1) = 2.10, *p* = 0.15). Thus, the LMM confirms a robust *condition* effect in terms of response magnitude but does not reveal group-dependent differences in mean dilation, consistent with the GAMM results indicating that the interaction between *group* and *condition* arises from differences in the temporal dynamics of the pupil response rather than from sustained amplitude differences.

**Hypothesis testing (GAMMs for correctly rejected targets vs. missed targets).** The best-fitting GAMM of BCPD (time-locked to the stimulus onset rather than the target onset) for correctly rejected non-targets vs. missed targets also retained a significant two-way interaction of *group* and *condition*. Model diagnostics confirmed appropriate convergence and smoothness estimation (all k-index values ≈ 0.98; all *p*-values ≥ 0.05). Model comparisons using maximum likelihood indicated that the model containing the interaction provided a significantly better fit than the null model (ΔAIC = −518.93, χ^2^(9) = 273.76 *p* < 0.001) as well as models including only *group* (ΔAIC = −518.29, χ^2^(6) = 262.34, *p* < 0.001) or only *condition* (ΔAIC = −91.85, χ^2^(6) = 57.98, *p* < 0.001). Both the *group*-only model (ΔAIC = 0.64, χ^2^(3) = 11.42, *p* < 0.001) and the *condition*-only model (ΔAIC = −427.08, χ^2^(3) = 215.78, *p* < 0.001) provided significantly better fits than the null model, although the *condition*-only model accounted for more variance than the *group*-only model (ΔAIC = −426.44, ML difference = 204.36). The model including the interaction outperformed a model including additive effects of *group* and *condition* (ΔAIC = −91.53; χ^2^(3) = 47.29, *p* < 0.001), indicating that the effects related to *group* and *condition* were not fully independent over time.

**Figure 5:**
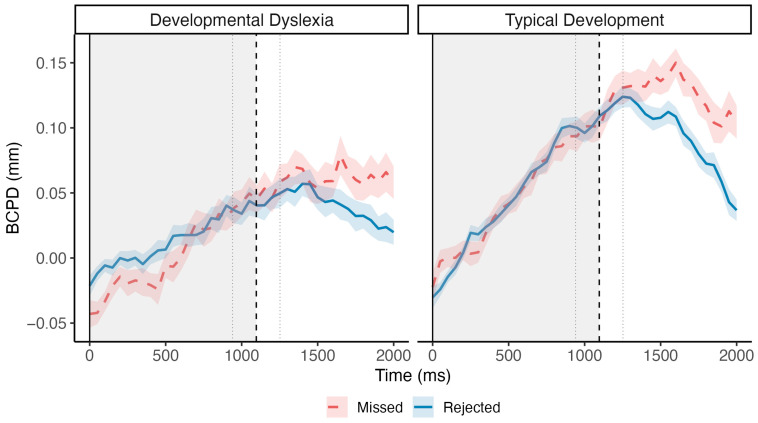
Time course of baseline-corrected pupil diameter (BCPD) for missed-target trials vs. correctly rejected non-target trials. The graph displays red curves for missed-target trials, blue curves for correctly rejected non-target trials. The left-hand panel shows results for participants with developmental dyslexia, the right-hand panel for typically developing participants. The x-axis is aligned to stimulus onset (0 ms). The solid vertical line marks stimulus onset, the dashed vertical line marks the average stimulus offset, and the dotted vertical lines represent ±1 standard deviation of the stimulus offset. The gray shaded area indicates the average stimulus duration.

Subsequent comparisons of the fitted curves (using plot_diff() function in R, [121,122]) identified larger pupil dilations in the TD group compared to the DD group across conditions. In addition, missed trials elicited higher pupil dilation than rejected trials in both groups. However, the significant divergence between missed and rejected curves emerged earlier in the TD group than in the DD group (before ~1500 ms), resulting in a longer temporal separation in TD. Peak analyses further clarified the source of this difference, indicating that it was specifically driven by latency differences for rejected trials, but not for missed trials. Missed trials showed comparable peak latencies across groups (TD = 1502 ms, BCPD = 0.146 mm; DD = 1511 ms, BCPD = 0.079 mm). In contrast, rejected trials peaked overall earlier than missed trials but later in the DD group than in the TD group (TD = 1293 ms, BCPD = 0.106 mm; DD = 1345 ms, BCPD = 0.053 mm). A visual inspection of the time-course trajectories (see Figure 5) confirmed this pattern and further highlighted that pupil dynamics of TD participants exhibited a clear and early temporal separation between missed and rejected responses, whereas in participants with DD, the difference emerged later and was less sustained.

Taken together, these findings indicate that participants with DD showed a reduced pupil dilation compared to TD participants across conditions, and that missed trials elicited higher and later peak activation than correctly rejected trials. Accordingly, the interaction between *group* and *condition* may derive from a latency shift specific to rejected trials in DD and a reduced differentiation between rejected and missed responses.

**Hypothesis testing (LMMs for correctly rejected targets vs. missed targets).** Additional LMM analyses based on BCPD scores averaged by participant and stimulus and extracted for each peak interval (i.e., 400 ms time window around the observed peak latency, cf. [50,129,130]) provided converging evidence for the GAMM results. The best-fitting LMM retained a significant contribution of *group* to mean pupil dilation across missed and rejected trials, with overall larger responses in the TD than in the DD group (*F*(1, 56.72) = 9.935, *p* = 0.003). In contrast, there was no evidence for a main effect of *condition* (*F*(1, 76.78) = 1.128, *p* = 0.291), and the interaction of *group* and *condition* did not improve model fit (*F*(1, 2146.40) = 0.211, *p* = 0.646). Moreover, including the three-way interaction among *group*, *condition*, and the *cognitive factor* did not significantly improve model fit (*F*(1, 2143.10) = 0.093, *p* = 0.761). Thus, the LMM aligns with the GAMM in confirming a robust group effect, but fails to capture the interaction, as time-averaged BCPD values reflect amplitude differences rather than temporal dynamics.

## 4. Discussion

The present study tested two hypotheses related to the phonological processing issues frequently observed in individuals with developmental dyslexia. According to the *Acoustic Sensitivity Hypothesis* [37,38,41,64], we expected that behavioral and pupil responses in children with DD would be reflective of a reduced perceptual sensitivity to the variability in acoustic salience. More specifically, the *Acoustic Sensitivity Hypothesis* predicted a negative relationship between phonological processing and pupil response, on the one hand, and acoustic salience, on the other hand. According to the *Cognitive Mediation Hypothesis* [29,30,31], we expected that behavioral and pupil responses in children with DD would be reflective of a limited cognitive support for phonological processing. More specifically, the *Cognitive Mediation Hypothesis* predicted a negative relationship between phonological processing and pupil response, on the one hand, and cognitive resources, on the other hand.

To investigate these hypotheses, we tested 28 children with DD and 29 typically developing peers using a phoneme monitoring task [80,82] and concurrent pupillometry [50]. In this task, participants had to detect specific phonemes (geminates vs. singletons; obstruents vs. sonorants) embedded in acoustically either more salient (i.e., strong) or less salient (i.e., weak) syllables of nonce-words, while pupillometry captured implicit responses to the phonological task. Pupil dilation has been previously reported in response to acoustic salience [51,55,60] or cognitive effort [56,59]. To test the *Acoustic Sensitivity Hypothesis*, we computed an acoustic salience index from measurements of F_0_, duration, and intensity, capturing the relative acoustic–prosodic weight of each phoneme within its context. To test the *Cognitive Mediation Hypothesis*, we derived an individual cognitive index by aggregating scores from participants’ performance in working memory, attentional shifting, and non-verbal reasoning tasks. The results first confirmed that on the behavioral level, Italian children with DD showed significantly lower sensitivity (d′) and accuracy in phoneme identification compared to their TD peers, driven primarily by a lower number of correctly identified (rather than correctly rejected) trials. Analyses of pupil responses indicated that, during correctly identified target trials, pupil dilation in the DD group was comparable in magnitude to that of TD children, albeit with a later dilation peak. In contrast, correctly rejected as well as missed trials revealed both significantly reduced pupil dilation and delayed peak latency in the DD group relative to the TD group. The key finding of the study is that behaviorally, the two groups differed in the number of correctly identified targets, while at the level of pupil responses, the two groups differed primarily in the magnitude and temporal dynamics in response to rejected or missed targets, which is quite noteworthy.

Notably, phoneme sensitivity (d′) significantly correlated with average pupil dilation during target processing (for both correctly identified targets and correctly rejected non-targets). This reinforces the interpretation of pupil size as a physiological marker of phonological processing, in line with previous findings that have shown pupil dilation to be sensitive to the demands of phonological processing [52,55,60]. Group-level differences in phoneme identification were not related to acoustic salience: this factor neither predicted accuracy, nor interacted with group, nor correlated with pupil dilation in either condition. Even though the cognitive factor did not emerge as a behavioral predictor, it significantly correlated with average pupil dilation during correctly rejected non-target trials for all participants. These findings indicate that variability in phoneme identification is potentially mediated by individual differences in cognitive abilities, such as IQ, working memory, and shifting attention [29,30,31]. Moreover, the effect may be more general than the dyslexic deficit idea tends to suggest [104].

Overall, the results of the current study do not provide direct support for the *Acoustic Sensitivity Hypothesis*. We found no clear evidence that variability in acoustic salience (as operationalized in the present study) significantly contributed to reduced phoneme identification in participants with DD. This is in line with a recent meta-analysis [47] suggesting that prosodic issues in DD may be task-dependent and come to light only in tasks which require an explicit, metalinguistic judgment. Whether perceptual reliance on acoustic salience is moderated by orthographic depth or the role of lexical stress in a language’s phonological system remains an open question for future research.

While the present findings also do not straightforwardly support the *Cognitive Mediation Hypothesis*, they provide an indication that phonological processing such as is required for a successful performance in the phoneme monitoring task of the present study leads to an increased cognitive effort in participants, not only during identification but also during rejection trials. Target selection and distractor inhibition are increasingly discussed and understood as partially distinct processes rather than outcomes of a single unified attentional mechanism [131,132,133,134,135]. The two operations may rely on separable neural substrates and draw on different cognitive resources [131,132,133,134,135]. In particular, distractor filtering has been shown to place additional demands on executive and working memory systems [135] and to involve inhibitory mechanisms that go beyond those engaged during target selection [132,134]. The presence of a large number of distractors in the present study may have incurred a relatively high filtering cost to all participants, with an especially negative effect on the identification of targets in the DD group. Consistent with this view, Marini et al. [132] demonstrated that proactive distraction filtering leads to behavioral costs, even in no-distractor trials, suggesting that anticipatory inhibition can interfere with concurrent target-related processing. Similarly, Sabri et al. [135] found that processing irrelevant stimuli consumes working memory resources, thereby limiting executive and attentional capacities required for the task-oriented focus.

Within the broader continuum of cognitive abilities of our participants, individuals with reduced cognitive abilities may require more time to integrate inhibitory and evaluative processes during distractor rejection, leading to delayed and attenuated pupillary responses. This account may also help to explain the more general latency shifts observed during target processing in DD, suggesting that increased executive demands can influence response timing across both non-target and target conditions. This interpretation is in line with pupillometric evidence showing that reduced cognitive availability (e.g., as that observed in older adults) tends to be associated with smaller or slower task-evoked pupil responses under cognitive load [57,136,137]. While we observed these patterns in our participants with DD, they should not be viewed as critical markers of the condition, but rather as reflecting gradations in cognitive functioning that interact with phonological processing [31]. We acknowledge, however, that this interpretation is post hoc and therefore preliminary, and dedicated future studies are needed to test it directly.

In summary, the present study contributes to the ongoing debate on the origin of phonological deficits in DD by providing evidence that cognitive resources, particularly inhibitory control, may play a stronger role in phonological processing than purely acoustic factors. Using a phoneme monitoring task combined with pupillometry, the study showed that children with DD were less accurate in identifying phonemes than their typically developing peers, yet this behavioral difference was not straightforwardly reflected in pupil responses to correctly identified targets. Rather, the two groups diverged most clearly in their physiological responses to missed targets and rejected distractors, suggesting that the locus of difficulty in this specific phonological task may be linked with the cognitive demands of distractor filtering and inhibitory control [132,134], with a downstream effect on target identification [132,135]. Given that target selection and distractor inhibition draw on partially distinct mechanisms and neural substrates [131,132,133,134,135], the filtering cost for children with DD may be so high that it constrains their resources available for target processing. Crucially, this dissociation between behavioral and physiological responses would not have been detectable from accuracy data alone, underscoring the value of pupillometry as a sensitive, real-time index of cognitive engagement during phonological processing [56,59]. Correlational analyses further indicated that individual cognitive profiles might be better predictors of pupillometric responses than a division into TD vs. DD group membership, suggesting a graded rather than categorical view of phonological processing in which performance reflects continuous variation in cognitive resources.

### Glossary of Key Terms (In Alphabetical Order)

- **Acoustic cues:** measurable acoustic properties of speech sounds (such as fundamental frequency, duration, and intensity) that contribute to the perception of contrasts within sound systems.
- **Acoustic salience:** The relative perceptual weight of a speech unit arising from the variation in acoustic cues within the unit’s context. In the present study, acoustic salience was operationalized as a composite factor including fundamental frequency, duration, and intensity.
- **Fundamental frequency (**F_0_**):** The lowest frequency component of a (quasi-)periodic sound, measured in Hz and perceived as pitch. In speech, it reflects the rate at which the vocal folds complete one full open–close cycle per second.
- **Lexical stress:** A phonological property of the mental lexicon, which gives a specific syllable (called a strong syllable) within a word more weight than the others (called weak syllables). In real speech, the property can be phonetically expressed through a number of (language-specific) acoustic cues, typically leading to an increase in acoustic salience of the corresponding syllable and to the perception of increased (local) prominence by a native listener.
- **Nonce-word:** a pronounceable word-like stimulus that does not exist in the mental lexicon.
- **Orthographic transparency/depth:** The degree to which sounds consistently and predictably correspond to orthographic symbols in a given writing system. Languages with shallow orthographies, such as Italian, have highly regular sound-to-letter mappings; languages with deep orthographies, such as English, do not. For historical reasons, some English letters have to be spelled but are not pronounced (e.g., *w* in *answer*); a single letter can map onto multiple phonemes (e.g., *<*a> in *bag*, *lake*, *was*, *raw* maps onto /æ/, /eɪ/, /ɒ/, /ɔ/) while the same phoneme can be spelled in multiple ways (e.g., */k/* in *calm*, *king*, *track*).
- **Phonological awareness:** the conscious ability to reflect on and manipulate the sound structures in one’s native language at multiple levels, including syllables, syllable constituents, and phonemes.
- **Phonological processing:** a set of mental operations involved in perceiving, accessing, identifying, discriminating, and using mental representations of speech sounds.
- **Phonological representations:** abstract mental encoding of linguistically relevant properties of speech sounds and syllable structures.
- **Speech prosody:** linguistically relevant properties of spoken language operating above the level of individual sounds and at the level of syllables, words, phrases, and utterances, typically encoded by acoustic variation in duration, loudness, and fundamental frequency, and conveying information such as lexical stress, phrasal accentuation and focus structure, syntactic phrasing, sentence mode, and conversational turn-taking.

## 5. Conclusions

The present study examined the role of acoustic salience and cognitive resources in phonological processing difficulties in developmental dyslexia, using a phoneme monitoring task with concurrent pupillometry. Children with DD showed lower phoneme identification accuracy than their TD peers, but pupil responses revealed a more nuanced picture: group differences emerged most clearly in physiological responses to rejected and missed trials rather than to correctly identified targets. While speech acoustics did not help to explain performance in either group, individual cognitive profiles were linked to pupil dilation especially during distractor rejection trials in the group of dyslexic children. These findings suggest that the cognitive demands of filtering irrelevant stimuli may place a disproportionate burden on children with DD and thus indirectly constrain their resources available for target identification. The results also demonstrate that pupillometry can help to uncover potential dissociations between phonological behaviours and related cognitive efforts, which behavioural data alone cannot reveal, and underscore the importance of incorporating physiological measures into the study of developmental dyslexia.

## Data Availability

Due to ethical restrictions, the datasets generated and analyzed for the purposes of the present study are available from the corresponding authors upon reasonable request.
